# Previous Use of Anti-Vascular Endothelial Growth Factor Receptor Agents Decreases Efficacy of Fruquintinib in Metastatic Colorectal Cancer Refractory to Standard Therapies

**DOI:** 10.3389/fonc.2020.587692

**Published:** 2020-11-13

**Authors:** Lei Wang, Huijiao Cao, Chang Jiang, Wenzhuo He, Yafei You, Kunwei Peng, Yanan Jin, Liangping Xia

**Affiliations:** Department of VIP Region, Sun Yat-sen University Cancer Center, State Key Laboratory of Oncology in South China, Collaborative Innovation Center for Cancer Medicine, Guangzhou, China

**Keywords:** metastatic colorectal cancer, third-line therapy, fruquintinib, neutrophil-lymphocyte ratio, survival outcome

## Abstract

**Purpose:**

Fruquintinib is an anti-vascular endothelial growth factor receptor (VEGFR) agent. The FRESCO trial demonstrated that patients with metastatic colorectal cancer (mCRC) refractory to standard therapies could benefit from fruquintinib with tolerable adverse events (AEs). However, the efficacy and safety of fruquintinib in clinical practice has scarcely been reported, especially in patients with previous use of anti-VEGFR agents.

**Methods:**

This retrospective study investigated the efficacy and safety of fruquintinib in patients with mCRC between January 2019 and December 2019. Progression-free survival (PFS) and overall survival (OS) were assessed by a Kaplan-Meier analysis and log-rank test. A Cox regression model was performed to identify independent prognostic factors.

**Results:**

A total of 46 patients were included. The median PFS and OS were 3.1 months (95% confidence interval [CI], 1.9–4.3 months) and 9.0 months (95% CI, 7.2–10.8 months), respectively. Patients previously treated with anti-VEGFR agents had shorter median PFS compared with those without previous use of anti-VEGFR agents (1.9 *vs.* 3.7 months, P = 0.006), while the median OS was similar between the two groups (8.5 *vs.* 9.0 months, P = 0.992). Multivariate analysis revealed that the neutrophil-lymphocyte ratio (NLR) was an independent prognostic factor in PFS (hazard ratio [HR], 2.230; 95% CI, 1.191–4.517, P = 0.014) and OS (HR, 4.221; 95% CI, 1.683–10.586; P = 0.002). The most common non-hematological and hematological AEs were hand-foot syndrome (37.0%) and anemia (39.1%), respectively.

**Conclusion:**

Fruquintinib was an effective third-line therapy in mCRC with tolerable AEs. Efficacy of fruquintinib was decreased in patients with previous use of anti-VEGFR agents. NLR was an independent prognostic factor in PFS and OS in patients treated with fruquintinib.

## Introduction

Colorectal cancer (CRC) is one of the most common causes of cancer-related deaths globally (1). Metastasis occurs in approximately 20% of newly diagnosed patients, and approximately 50% of early stage CRC develop metastasis (2). Although cytotoxic drugs combined with targeted agents are standard first- and second-line therapies for metastatic CRC (mCRC) in National Comprehensive Cancer Network (NCCN) guidelines (3), patients still experience disease progression after standard treatment. The third-line therapy in mCRC has not been well-established, although regorafenib and trifluridine/tipiracil (TAS-102) are available. Regorafenib is an inhibitor targeting vascular endothelial growth factor receptor (VEGFR), which has an anti-angiogenic function (4). Both the CORRECT (5) and CONCUR (6) trials observed improved median progression-free survival (PFS) and overall survival (OS) in patients treated with regorafenib compared with those treated with placebo. TAS-102 is an orally administered combined chemotherapy agent. The RECOURSE (7) and TERRA (8) trials revealed a survival benefit of TAS-102 compared with placebo in mCRC refractory to standard therapies.

However, disease control of mCRC refractory to standard therapies is still limited. As a result, the anti-VEGFR tyrosine kinase inhibitor (TKI), fruquintinib, has been investigated (9). A phase Ib study and a randomized double-blind phase II study showed a significant improvement of PFS in the fruquintinib group compared with the placebo group (P < 0.001) in patients with treatment-refractory mCRC (10). In the FRESCO trial, 278 patients were randomized to the fruquintinib group and 138 patients to the placebo group. The median PFS and OS were significantly improved in the fruquintinib group compared with the placebo group (3.7 *vs.* 1.8 months, P < 0.001; and 9.3 *vs.* 6.6 months, P < 0.001; respectively), and treatment-related adverse events (AEs) in the fruquintinib group were tolerable (11). Therefore, fruquintinib was approved in China in September 2018 and in the United States in June 2020 (12).

The association between inflammatory and immune status, including neutrophil-lymphocyte ratio (NLR) and platelet-lymphocyte ratio (PLR), and the efficacy of anti-VEGFR therapy has also been investigated. Santoni and colleagues (13) evaluated the prognostic role of NLR in patients treated with anti-VEGFR therapy in metastatic renal cell carcinoma and revealed that NLR was an independent prognostic factor for both OS (P < 0.001) and PFS (P = 0.03). Moreover, Hu et al. (14) conducted a prospective study assessing the efficacy of regorafenib in metastatic gastrointestinal stromal tumors in a Taiwanese population, and the results suggested that high NLR and PLR predicted unfavorable OS (P = 0.033 and P = 0.019, respectively). However, the prognostic role of NLR and PLR in treatment-refractory mCRC treated with fruquintinib has rarely been explored.

Clinical practice is complex in mCRC refractory to standard therapies. Although clinical trials revealed notable efficacy and tolerance of fruquintinib, patients with previous anti-VEGFR agents were excluded. Therefore, the efficacy and safety of fruquintinib require further assessment. It remains unclear whether NLR and PLR can predict fruquintinib efficacy. Thus, this retrospective study was conducted to provide insight for clinical practice.

## Methods and Materials

### Patient Selection

In this retrospective study, patients with mCRC between January 2019 and December 2019 at Sun Yat-sen University Cancer Center were reviewed. The inclusion criteria were as follows: (1) CRC with metastasis; (2) refractory to at least two lines of standard systemic therapies; and (3) received at least one dose of fruquintinib. The exclusion criteria were as follows: (1) combination therapy of fruquintinib with other anti-tumor drugs; (2) lack of treatment data; and (3) lost follow-up. All clinical records, image information, and blood profiles were reviewed. The study was approved by the Medical Ethics Committee of Sun Yat-sen University Cancer Center (B2020-256). Key data of this study has been uploaded onto the Research Data Deposit public platform (http://www.researchdata.org.cn), with approval number of RDDA2020001709.

### Statistical Analysis

NLR was categorized as ≤3 and >3 (15). PLR was categorized as <150, 150–300, and >300 (16). Tumor response was defined by the Response Evaluation Criteria in Solid Tumors version 1.1 (17). The objective response rate (ORR) referred to the rate of complete response (CR) and partial response (PR), and the disease control rate (DCR) referred to the rate of CR, PR, and stable disease (SD). PFS was defined as the beginning of fruquintinib treatment to disease progression or death, and OS was defined as the beginning of fruquintinib treatment to death from any cause. AEs during treatment were assessed based on the Common Terminology Criteria for Adverse Events version 3.0 (18).

Continuous and categorical variables were compared by chi-squared and Mann-Whitney U tests, respectively. Survival outcomes were evaluated by the Kaplan-Meier method and log-rank test. A Cox regression model was performed to identify independent prognostic factors. All tests were two-sided and P < 0.05 was considered statistically significant. Statistical analyses were performed using SPSS 21.0 software.

## Results

### Patient Characteristics

A total of 46 mCRC patients treated with fruquintinib monotherapy were identified (**Table 1**). The median age was 59 years (range, 21–85 years), and 60.9% of patients were male. Four of the 46 (8.7%) patients had an Eastern Cooperative Oncology Group Performance Status (ECOG PS) of 2. The most common primary site was the colon (71.7%), followed by the rectum (17.4%). More than half of the patients had metastatic lesions in more than two organs (58.7%) and the incidence of the RAS mutation was 54.3%. Moreover, 38/46 (82.6%) patients were previously treated with bevacizumab, and 14/46 (30.4%) patients had previously been treated with anti-VEGFR agents (8 of regorafenib alone; 3 of apatinib alone; and 3 of both regorafenib and apatinib). Baseline characteristics between the 14 patients previously treated with anti-VEGFR agents and 32 patients not treated with anti-VEGFR agents were well balanced except for previous lines of therapy (**Supplementary Table**).

**Table 1:** Baseline characteristics of 46 patients treated with fruquintinib.

Characteristics	Number
Age (years)	
<60	25 (54.3)
≥60	21 (45.7)
Gender	
Male	28 (60.9)
Female	18 (39.1)
ECOG PS	
0–1	42 (91.3)
2	4 (8.7)
Primary site	
Colon	33 (71.7)
Rectum	8 (17.4)
Unknown	5 (10.9)
Metastatic organs	
1–2	19 (41.3)
≥3	27 (58.7)
RAS mutant	
Yes	25 (54.3)
No	12 (26.1)
Unknown	9 (19.6)
Lines of previous therapy	
2	33 (71.7)
≥3	13 (28.3)
Previous anti-tumor agents	
Fluoropyromidine	45 (97.8)
Irinotecan	42 (91.3)
Oxaliplatin	43 (93.5)
Bevacizumab	38 (82.6)
Cetuximab	13 (28.3)
Anti-VEGFR	14 (30.4)

VEGFR, vascular endothelial growth factor receptor; ECOG PS, Eastern Cooperative Oncology Group performance status.

### Treatment

A total of 43 patients were initially treated with 5 mg per day with a 28-day treatment cycle (3 weeks on/1 week off). Three patients had an initially reduced dose of 4 mg (two patients) and 3 mg (one patient) according to the judgment of clinicians. Six (13.0%) patients experienced dose reduction, 12 (26.1%) patients had treatment interruption, and 6 (13.0%) patients discontinued the therapy. Therapies after fruquintinib were as follows: rechallenge of chemotherapy with or without bevacizumab in 16 (34.8%) patients and local therapy in 4 (8.7%) patients.

### Efficacy

**Figure 1:**
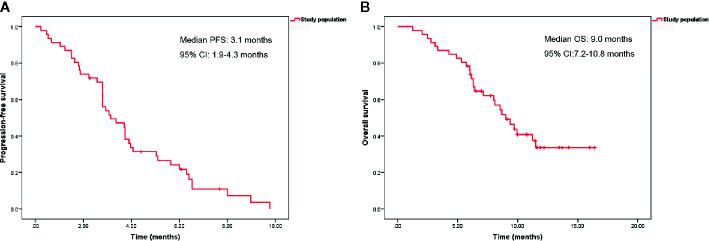
Kaplan-Meier curves of progression-free survival **(A)** and overall survival **(B)** in 46 patients.

**Figure 2:**
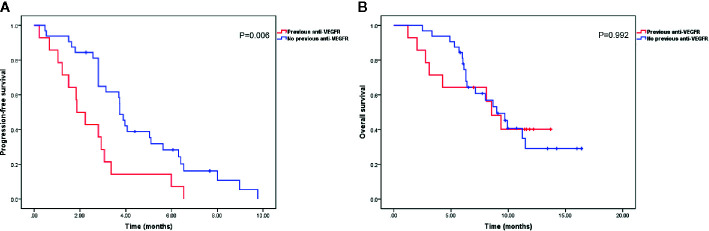
Kaplan-Meier curves of progression-free survival **(A)** and overall survival **(B)** in patients with or without previous anti-vascular endothelial growth factor receptor (VEGFR) agent treatment.

With a median follow-up time of 9.7 months (range, 1.2–16.4 months), the median PFS and OS were 3.1 months (95% confidence interval [CI], 1.9–4.3 months) and 9.0 months (95% CI, 7.2–10.8 months), respectively (**Figure 1**). The ORR and DCR rates were 4.9 and 51.2%, respectively. Patients previously treated with anti-VEGFR agents had shorter median PFS than patients not previously treated with anti-VEGFR agents (1.9 months [95% CI, 1.1–2.6 months] *vs.* 3.7 months [95% CI, 3.4–4.1 months], P = 0.006). However, no significant difference was observed in median OS between the two groups (9.0 months [95% CI, 6.8–11.2 months] *vs.* 8.5 months [95% CI, 6.4–10.7 months], P = 0.992) (**Figure 2**).

### Univariate and Multivariate Analyses

Univariate and multivariate analyses were performed to identify independent prognostic factors (**Tables 2** and **3**). The univariate analysis revealed that previous anti-VEGFR agents (hazard ratio [HR], 2.423; 95% CI, 1.245–4.715; P = 0.009), NLR (HR, 1.976; 95% CI, 1.061–3.682; P = 0.032), and hand-foot syndrome (HFS) (HR, 2.153; 95% CI, 1.077–4.304; P = 0.030) were significantly associated with PFS. NLR (HR, 2.332; 95% CI, 1.085–5.011; P = 0.030) was significantly associated with OS. Multivariate analysis demonstrated that previous anti-VEGFR therapy (HR, 2.021; 95% CI, 1.009–4.074; P = 0.047) and elevated NLR (HR, 2.230; 95% CI, 1.191–4.517; P = 0.014) were independent prognostic factors in PFS. Moreover, elevated NLR (HR, 4.221; 95% CI, 1.683–10.586; P = 0.002) was an independent prognostic factor in OS.

**Table 2:** Univariate analysis of PFS and OS in 46 patients.

Variables	PFS		OS	
	Univariate analysis		Univariate analysis	
	HR (95% CI)	P	HR (95% CI)	P
Age (years)				
<60	Ref.		Ref.	
≥60	1.362 (0.734–2.527)	0.328	1.036 (0.486–2.210)	0.926
Gender				
Male	Ref.		Ref.	
Female	1.048 (0.556–1.974)	0.886	1.273 (0.589–2.752)	0.540
ECOG PS				
0–1	Ref.		Ref.	
2	1.240 (0.377–4.081)	0.723	1.061 (0.251–4.491)	0.936
Primary site				
Colon	Ref.		Ref.	
Rectum	0.509 (0.168–1.541)	0.232	0.875 (0.333–2.305)	0.788
Unknown	0.615 (0.163–2.325)	0.474	0.668 (0.207–2.157)	0.500
Metastatic organs				
1–2	Ref.		Ref.	
≥3	1.703 (0.886–3.275)	0.110	1.993 (0.134–7.376)	0.398
RAS mutant				
Yes	Ref.		Ref.	
No	1.225 (0.518–2.896)	0.644	1.494 (0.494–4.519)	0.477
Unknown	1.805 (0.690–4.723)	0.229	1.867 (0.559–6.232)	0.310
Lines of previous therapy				
2	Ref.		Ref.	
≥3	1.187 (0.589–2.392)	0.631	1.830 (0.691–4.844)	0.188
Previous Bevacizumab				
Yes	Ref.		Ref.	
No	1.017 (0.446–2.319)	0.968	2.659 (0.997–7.091)	0.051
Previous anti-VEGFR				
Yes	Ref.		Ref.	
No	2.423 (1.245–4.715)	0.009	1.004 (0.438–2.303)	0.992
Sufficient Treatment^*^				
Yes	Ref.		Ref.	
No	1.093 (0.584–2.045)	0.782	1.236 (0.577–2.650)	0.586
NLR				
≤3	Ref.		Ref.	
>3	1.976 (1.061–3.682)	0.032	2.332 (1.085–5.011)	0.030
PLR				
>300	Ref.		Ref.	
150–300	0.659 (0.283–1.534)	0.333	1.122 (0.425–2.962)	0.816
<150	0.570 (0.234–1.385)	0.214	0.396 (0.137–1.147)	0.088
CEA				
≤5	Ref.		Ref.	
>5	1.308 (0.506–3.382)	0.579	1.072 (0.321–3.577)	0.910
Hand-foot syndrome				
Yes	Ref.		Ref.	
No	2.153 (1.077–4.304)	0.030	1.262 (0.565–2.814)	0.570
Hypertension				
Yes	Ref.		Ref.	
No	1.796 (0.869–1.796)	0.114	1.291 (0.545–3.060)	0.562
Fatigue				
Yes	Ref.		Ref.	
No	1.128 (0.544–2.340)	0.746	1.987 (0.750–5.264)	0.167

PFS, progression-free survival; OS, overall survival; HR, hazard ratio; CI, confidence interval; ECOG PS, Eastern Cooperative Oncology Group performance status; VEGFR, vascular endothelial growth factor receptor; NLR, neutrophil-to-lymphocyte ratio; PLR, platelet-to-lymphocyte ratio, CEA, carcinoembryonic antigen.

^*^Fruquintinib was administered without dose reduction, treatment interruption, or therapy discontinuation.

**Table 3:** Multivariate analysis of PFS and OS in 46 patients.

Variables	PFS		OS	
	Multivariate analysis		Multivariate analysis	
	HR (95% CI)	P	HR (95% CI)	P
Metastatic organs				
1–2	Ref.			
≥3	1.701 (0.814–3.552)	0.143		
Lines of previous therapy				
2			Ref.	
≥3			1.745 (0.649–4.695)	0.270
Previous Bevacizumab				
Yes			Ref.	
No			2.458 (0.906–6.673)	0.078
Previous anti-VEGFR				
Yes	Ref.			
No	2.021 (1.009–4.074)	**0.047**		
NLR				
≤3	Ref.		Ref.	
>3	2.320 (1.191–4.517)	**0.014**	4.221 (1.683–10.586)	**0.002**
PLR				
>300			Ref.	
150–300			2.295 (0.920–9.296)	0.771
<150			0.836 (0.252–2.780)	0.069
Hand-foot syndrome				
Yes	Ref.			
No	1.807 (0.703–4.462)	0.219		
Hypertension				
Yes	Ref.			
No	1.005 (0.664–1.521)	0.920		
Fatigue				
Yes			Ref.	
No			2.153 (0.759–6.108)	0.149

PFS, progression-free survival; OS, overall survival; HR, hazard ratio; CI, confidence interval; ECOG PS, Eastern Cooperative Oncology Group performance status; VEGFR, vascular endothelial growth factor receptor; NLR, neutrophil-lymphocyte ratio; PLR, platelet-lymphocyte ratio; CEA, carcinoembryonic antigen.Bold values represent the P-values that are statistically significant.

### Subgroup Analysis

Since patients with elevated NLR were likely to have poor survival outcomes, we investigated whether NLR was able to predict survival outcomes in patients with or without previous treatment with anti-VEGFR agents (**Table 4**). In the previous anti-VEGFR group, patients with NLR >3 had shorter median PFS compared with patients with NLR ≤3 (1.8 *vs.* 3.4 months; P = 0.026). In the no previous anti-VEGFR group, patients with NLR >3 had shorter median OS compared with patients with NLR ≤3 (6.0 *vs.* 11.5 months; P = 0.003).

**Table 4:** Survival outcomes in patients with or without previous anti-VEGFR agents stratified by NLR.

Variables	Median PFS (months, 95% CI)			Median OS (months, 95% CI)		
	NLR ≤3	NLR >3	P	NLR ≤3	NLR >3	P
Previous anti-VEGFR	3.4 (2.4–4.3)	1.8 (0.9–2.8)	0.026	9.4 (6.7–12.1)	8.5 (6.4–10.7)	0.751
No previous anti-VEGFR	4.0 (3.5–4.4)	3.7 (3.4–4.1)	0.365	11.5 (8.7–14.3)	6.0 (5.0–7.1)	0.003

PFS, progression-free survival; OS, overall survival; CI, confidence interval; VEGFR, vascular endothelial growth factor receptor; NLR, neutrophil-lymphocyte ratio.

### Safety

AEs during treatment are listed in **Table 5**. The most common non-hematological AEs were HFS (37.0%), hepatotoxicity (32.6%), and hypertension (28.3%), while the most common ≥ Grade 3 AEs were HFS (13.0%), hypertension (6.5%), and hepatotoxicity (4.3%). Diarrhea and proteinuria occurred in 13.0 and 6.5% patients, respectively, with no patients suffering from these two AEs above Grade 2. Fatigue affected 26.1% of patients with 1 patient experiencing ≥ Grade 3 fatigue. Two patients experienced Grade 1 bleeding. The most common hematological AEs was anemia (39.1%), while the most common ≥ Grade 3 hematological AEs was thrombocytopenia (8.7%). No treatment-related death occurred. The most common AEs related to dose reduction was HFS (3/6, 50.0%), while the leading three causes of treatment interruption were thrombocytopenia (4/12, 33.3%), HFS (3/12, 25.0%), and proteinuria (2/12, 16.7%). In addition, fruquintinib discontinuation was observed in six patients due to HFS (3/6, 50%), proteinuria (1/6, 16.7%), and patients’ own reasons (2/6, 33.3%).

**Table 5:** Adverse events of 46 patients treated with fruquintinib.

Adverse events	Any grade	Grade ≥3
Non-hematologic		
Hypertension	13 (28.3)	3 (6.5)
Hand-foot syndrome	17 (37.0)	6 (13.0)
Proteinuria	3 (6.5)	0 (0.0)
Hepatotoxicity	15 (32.6)	2 (4.3)
Fatigue	12 (26.1)	1 (2.2)
Bleeding	2 (4.3)	0 (0.0)
Diarrhea	6 (13.0)	0 (0.0)
Hematologic		
Leukopenia	6 (13.0)	2 (4.3)
Neutropenia	5 (10.9)	1 (2.2)
Thrombocytopenia	11 (23.9)	4 (8.7)
Anemia	18 (39.1)	2 (4.3)

## Discussion

Angiogenesis plays a crucial role in tumor growth, because the tumor-associated neovasculature supplies oxygen and nutrients to support tumor cell survival (19). Since Kim et al. (20) found that anti-vascular endothelial growth factor (VEGF) antibodies impaired neovascularization and tumor growth in mice, VEGF/VEGFR inhibitors have been widely explored in various advanced cancers (21). As treatment after first- and second-line therapies in mCRC is limited, the use of VEGFR inhibitors has been investigated. In the CORRECT trial (5), the median PFS and OS were improved in the regorafenib group compared with the placebo group (1.9 *vs.* 1.7 months, P < 0.0001; and 6.4 *vs.* 5.0 months, P = 0.0052; respectively) in mCRC refractory to at least two line standard therapies. These results were confirmed in the CONCUR trial (median PFS: 3.2 *vs.* 1.7 months, P < 0.0001; median OS: 8.8 *vs.* 6.3 months, P = 0.00016; respectively) (6). A recent prospective study of apatinib also showed efficacy in chemotherapy-refractory mCRC with median PFS and OS of 4.8 months (95% CI, 3.653–5.887 months) and 9.1 months (95% CI, 5.155–13.045 months), respectively (22). In the present study, we retrospectively explored the efficacy of the newly approved VEGFR inhibitor, fruquintinib, and observed shorter median PFS (3.1 *vs.* 3.7 months) and OS (8.6 *vs.* 9.3 months) compared with the FRESCO trial. The DCR rate was also lower in the present study compared with that in the FRESCO trial (51.2 *vs.* 62.2%).

The results of this retrospective study could be interpreted in several aspects. First, bevacizumab combined with chemotherapy is recommended as standard therapy for mCRC (3), but the impact of prior bevacizumab on later treatment is still unclear. Retrospective and prospective studies (22, 23) regarding the efficacy of apatinib in mCRC revealed no significant differences between patients with or without prior treatment with bevacizumab in PFS and OS. Besides, clinical trials (10, 11) evaluating the efficacy of fruquintinib did not exclude patients with previous use of bevacizumab. In the present study, 82.6% of patients had received bevacizumab previously, and multivariate analysis showed that previous use of bevacizumab had a non-statistically significant impact on OS (P = 0.078) in treatment-refractory mCRC treated with fruquintinib. This was consistent with previous results, indicating that different mechanisms might exist between VEGF and VEGFR inhibitors in suppressing tumor growth, resulting in scarce cross-resistance. However, the underlying mechanism is still unclear, and further studies are expected. Second, we observed poor median PFS in patients previously treated with anti-VEGFR agents (regorafenib and apatinib). Regorafenib is an anti-VEGFR TKI that inhibits angiogenesis (VEGFR-1, -2, -3, and TIE2) and oncogenic receptor tyrosine kinases (4). Apatinib is an anti-VEGFR TKI that targets VEGFR-2, as well as c-KIT, RET, and c-SRC (24). Fruquintinib is an anti-VEGFR TKI that inhibits VEGFR-1, -2, and -3 (25). Thus, we postulated that prior regorafenib and apatinib might decrease the efficacy of fruquintinib because of their overlapping functions, leading to shorter median PFS. These results suggested that fruquintinib might not be a good choice following treatment with anti-VEGFR agents. The current NCCN guidelines recommend ramucirumab combined with FOLFIRI (folinic acid, 5-fluorouracil, and irinotecan) for the treatment of mCRC with disease progression after previous oxaliplatin based therapy without irinotecan (3). Moreover, the REVERCE study (26) reported longer median OS with regorafenib followed by cetuximab ± irinotecan rather than cetuximab ± irinotecan followed by regorafenib (17.4 *vs.* 11.6 months; P = 0.0293) in KRAS exon 2 wild-type mCRC after failure of fluoropyrimidine, oxaliplatin, and irinotecan. With further investigations of anti-VEGFR therapy in mCRC as first- or second-line therapy, the potential use of fruquintinib requires further study. We are thus anticipating the results of two clinical trials, a global phase III trial (NCT04322539) investigating efficacy and safety of fruquintinib in patients with refractory mCRC, and a phase II trial (NCT04296019) exploring efficacy and safety of fruquintinib as a maintenance therapy following first-line treatment for mCRC. On the other hand, mCRC is molecularly heterogeneous with various biomarkers predicting response to treatment, such as RAS and BRAF mutations. This leads to significant challenges in planning an optimal treatment strategy for the refractory population (27). Clinical trials have explored the possibility of rechallenge of chemotherapy with or without targeted drugs such as cetuximab or bevacizumab (28–30), combination therapy including anti-BRAF and anti-HER2 agents according to BRAF mutation and HER2 amplification status (31, 32), and interventional techniques for local disease (33) in treatment-refractory mCRC. These explorations reported promising efficacy and safety, although high-quality evidence is still limited. Therefore, treatment strategies based on biomarker examination and disease evaluation might be the future direction for the refractory population. Third, we observed statistical significance of HFS in PFS in univariate analysis (HR, 2.153; 95% CI, 1.077–4.304; P = 0.030). A previous meta-analysis reported that VEGFR TKIs increased the risk of HFS (P < 0.00001) (34). Studies have indicated that HFS might result from inhibition of targeted receptors in healthy tissue by anti-angiogenic agents, revealing potential effectiveness of VEGF/VEGFR blockade (35, 36). Thus, we proposed that the presence of HFS might be an indicator of effectiveness of fruquintinib, and further studies with large sample size are expected. Fourth, the predictive potential of systemic inflammation in CRC has been widely investigated (37). Neutrophils are a major component of peripheral blood and are considered inflammatory cells. They are engaged in supporting adaptive immunity yielding signaling molecules that include the angiogenetic growth factor VEGF (38). Lymphocytes are considered an important part of anti-tumor immunity leading to cytotoxic cell death. High neutrophils or low lymphocytes in the peripheral circulation may result in a reduced immunological response, which can weaken treatment efficacy and lead to poor survival outcomes. In the present study, we identified NLR as an independent prognostic factor in PFS and OS among patients treated with fruquintinib, consistent with previous results (13, 14). Fifth, patients in our cohort showed shorter OS compared with the FRESCO trial. We prefer to attribute these results to older patients with poor performance status as well as the presence of metastatic lesions in multiple organs in the patients enrolled in our study.

The AEs profile in our study had some differences compared with that in the FRESCO trial (11). Hypertension (55.4%) was the most common side effect in the FRESCO trial, while HFS (37.0%) was the most common AE in our cohort. We prefer to attribute this difference to outpatient treatment, resulting in unsatisfactory observation of hypertension. In addition, the incidence of hematologic AEs was frequent in the present study. Anemia, which was not reported in the FRESCO trial, had a high rate of 39.1% in our cohort, and 8.7% patients experienced ≥ Grade 3 thrombocytopenia. This might result from heavy previous treatment (lines of previous therapy ≥3, 58.7%) and poor performance status (ECOG PS = 2, 8.7%) prior to treatment with fruquintinib.

The present study also had limitations. This was a retrospective study from a single institution with a small sample size. Although bias was unavoidable, we collected detailed data to reveal real-world treatment of fruquintinib monotherapy in mCRC refractory to standard therapies.

## Conclusion

Our study demonstrated that fruquintinib was effective as a third-line therapy for mCRC refractory to standard therapies with tolerable AEs. The benefit of fruquintinib in patients previously treated with anti-VEGFR agents was decreased. In addition, NLR was an independent prognostic factor in the efficacy of fruquintinib. Because this is a retrospective study with a small sample size from a single institution, further prospective investigations are warranted.

## Data Availability Statement

The raw data supporting the conclusions of this article will be made available by the authors, without undue reservation.

## Ethics Statement

The studies involving human participants were reviewed and approved by the Medical Ethics Committee of Sun Yat-sen University Cancer Center. The patients/participants provided their written informed consent to participate in this study. Written informed consent was obtained from the individual(s) for the publication of any potentially identifiable images or data included in this article.

## Author Contributions

LX: concept and design. LW, HC: data collection and statistical analysis. LW, HC, CJ, WH: results interpretation and manuscript writing. YY, KP, YJ: manuscript revision. All authors contributed to the article and approved the submitted version.

## Conflict of Interest

The authors declare that the research was conducted in the absence of any commercial or financial relationships that could be construed as a potential conflict of interest.

## Abbreviations

CRC, colorectal cancer; mCRC, metastatic colorectal cancer; NCCN, National Comprehensive Cancer Network; TAS-102, trifluridine/tipracil; VEGFR, vascular endothelial growth factor receptor; VEGF, vascular endothelial growth factor; TKI, tyrosine kinase inhibitor; AE, adverse event; PFS, progression-free survival; OS, overall survival; ECOG PS, Eastern Cooperative Oncology Group performance status; CR, complete response; PR, partial response; SD, stable disease; PD, progressive disease; ORR, objective response; DCR, disease control rate; CI, confidence interval; HR, hazard ratio; NLR, neutrophil-lymphocyte ratio; PLR, platelet-lymphocyte ratio; CEA, carcinoembryonic antigen; HFS, hand-foot syndrome.
