# Supplementary material for: Previous Use of Anti-Vascular Endothelial Growth Factor Receptor Agents Decreases Efficacy of Fruquintinib in Metastatic Colorectal Cancer Refractory to Standard Therapies
Source: Front Oncol. 2020 Nov 13;10:587692. doi: 10.3389/fonc.2020.587692 (PMC7691567; doi:10.3389/fonc.2020.587692)
Supplement: Supplementary file 1 [file Table_1.docx]

Baseline characteristics of patients with or without previous anti-VEGFR

| Characteristics | Previous anti-VEGFR  (N=14) | No previous anti-VEGFR  (N=32) | P |
| --- | --- | --- | --- |
| Age (years) |  |  | 0.128 |
| <60 | 10 (71.4) | 15 (46.9) |  |
| ≥60 | 4 (28.6) | 17 (53.1) |  |
| Gender |  |  | 0.323 |
| Male | 7 (50.0) | 21 (65.6) |  |
| Female | 7 (50.0) | 11 (34.4) |  |
| ECOG PS |  |  | 0.397 |
| 0-1 | 13 (92.9) | 29 (90.6) |  |
| 2 | 1 (7.1) | 3 (9.4) |  |
| Primary site |  |  | 0.373 |
| Colon | 11 (78.6) | 22 (68.8) |  |
| Rectum | 3 (21.4) | 5 (15.6) |  |
| Unknown | 0 (0.0) | 5 (15.6) |  |
| Metastatic organs |  |  | 0.614 |
| 1-2 | 5 (35.7) | 14 (43.8) |  |
| ≥3 | 9 (64.3) | 18 (56.3) |  |
| RAS mutant |  |  | 0.874 |
| Yes | 8 (57.1) | 17 (53.1) |  |
| No | 2 (14.3) | 10 (31.3) |  |
| Unknown | 4 (28.6) | 5 (15.6) |  |
| Lines of previous therapy |  |  | 0.004 |
| 2 | 6 (42.9) | 27 (84.4) |  |
| ≥3 | 8 (57.1) | 5 (15.6) |  |
| Previous anti-tumor agents |  |  |  |
| Fluoropyromidine | 14 (100.0) | 31 (96.9) | 0.508 |
| Irinotecan | 14 (100.0) | 25 (87.5) | 0.060 |
| Oxaliplatin | 13 (92.9) | 30 (93.8) | 0.911 |
| Bevacizumab | 12 (85.7) | 26 (81.3) | 0.716 |
| Cetuximab | 4 (28.6) | 9 (28.1) | 0.976 |

VEGFR, vascular endothelial growth factor receptor; ECOG PS, Eastern Cooperative Oncology Group performance status.
